# Investigation of the Cognitive Activity Between the Leaf Extracts of Eclipta alba and Ziziphus jujuba in Diabetic Animal Models

**DOI:** 10.7759/cureus.55400

**Published:** 2024-03-02

**Authors:** Eliya Raju Annepaka, Muthulakshmi Rangasmy, Srilakshmi Panakala, Suresh Babu Sayana

**Affiliations:** 1 Physiology, Meenakshi Medical College Hospital and Research Institute, Meenakshi Academy of Higher Education and Research (MAHER) University, Chennai, IND; 2 Biochemistry, Mamata Medical College, Khammam, IND; 3 Pharmacology, Government Medical College and General Hospital, Baramati, IND

**Keywords:** antidiabetic, ziziphus jujuba, glibenclamide, cognitive activity, eclipta alba

## Abstract

Diabetes mellitus (DM), a prevalent metabolic disorder, is associated with widespread damage to bodily systems, notably causing significant dysfunction within the peripheral and central nervous systems (CNS). The primary objective of this study is to explore the extent of DM's impact on cognitive and behavioral functions and to evaluate the therapeutic potential of ethanol leaf extracts from *Ziziphus jujuba* (ZJ) and *Eclipta alba* (EA) in mitigating these adverse effects. Utilizing an established animal model, we aimed to determine the effectiveness of these plant extracts in ameliorating the cognitive impairments commonly seen in diabetic states.

In our experimental framework, we allocated Wistar rats (n=6 per group) into eight different groups, inducing DM through alloxan administration. The intervention groups were treated orally with either the standard antidiabetic drug glibenclamide or varying doses of ZJ and EA extracts over periods of seven and 21 days. Throughout the study, we carefully tracked fluctuations in blood glucose levels, noting considerable decreases, particularly following the 21-day treatment interval.

Post-treatment, the rats' cognitive functions were assessed using the Morris water maze (MWM) test. This evaluation revealed significant cognitive enhancement in the diabetic rats administered with ZJ and EA extracts, with these groups displaying reduced latency in finding the submerged platform, indicative of improved learning and memory. These observations were statistically significant (p<0.01).

The findings underscore the hypoglycemic effects of ZJ and EA extracts and suggest their viability as cognitive enhancers in the context of DM. The protective effects of these extracts against cognitive decline caused by DM are clear. They add important new information to the research on natural phytochemicals for managing chronic diseases. This study opens new avenues for the application of these substances in treating neurocognitive disorders associated with DM.

## Introduction

The current global prevalence of diabetes mellitus (DM) and its associated metabolic complications has reached epidemic proportions. DM is a complex metabolic disorder characterized by an inefficient metabolism of proteins, fats, and carbohydrates, primarily due to either a deficiency in insulin production or ineffective insulin action [1-3]. One of the critical consequences of DM is hyperglycemia, which causes extensive damage to various bodily systems, particularly brain cells. This damage is closely associated with the development of neurodegenerative diseases, leading to a progressive decline in memory and cognitive function, hallmarks of dementia [1-6].

In countries like China and India, there is a profound tradition and extensive knowledge base in phytomedicine. The unique and varied properties of natural substances have long captivated chemists and biologists. Research indicates that approximately half of all approved pharmaceutical drugs are derived from natural sources. Recently, there has been a surge in interest in plant-based natural compounds, such as flavonoids, terpenoids, and steroids, due to their wide range of pharmacological properties, including antidiabetic and nootropic effects.

*Eclipta alba* (EA), commonly known as "bhringraj" and a member of the Asteraceae family, is a globally prevalent herb found in regions such as Brazil, China, India, and Nepal. Extensive research on EA has primarily focused on its hair growth-promoting capabilities. Its extract exhibits notable antibacterial, anti-inflammatory, and hepatoprotective effects, especially when challenged with carbon tetrachloride (CCl4) [7]. The hydroalcoholic extract of EA is also recognised for its anti-malarial and anti-cancer properties, underscoring the plant's extensive therapeutic potential. It is well known that *Eclipta prostrata* (EP) has antioxidant properties. Studies have shown that it can help make prostaglandins and has strong antioxidant activity, which was seen in extracts of ethanol, hexane, water, and ethyl acetate [7]. In the context of DM treatment, plants that produce chemicals capable of inhibiting the conversion of carbohydrates to glucose show great promise. For example, ecclalbasaponin II, a methanol extract from EP, lowered blood sugar levels in rats that had been given alloxan to cause DM [8]. Putting EA with other herbs, like in the Pan-five formula, has been shown to help with DM and water retention by restoring cell function and pancreatic regeneration [8]. The ethanolic leaf extract of EA has many chemicals in it, including sugars, lactones, steroids, terpenoids, glycosides, esters, flavonoids, and tannins. These chemicals can all help prevent DM and fix problems that happen in mice that were given alloxan to make them diabetic [9,10].

*Ziziphus jujuba* (ZJ), originally from Central Asia, has spread across Asia, Europe, and North America, with naturalised populations found in various locations. This species is also cultivated in private gardens and on integrated organic farms [11,12]. Recent phytochemical research has verified the anti-inflammatory, antioxidant, immunostimulating, hepatoprotective, anti-obesity, anti-cancer, and gastrointestinal protective properties of ZJ [13]. The antioxidant activity of ZJ aqueous extracts has been empirically proven.

Thus, this study explores the effects of EA and ZJ on cognitive function after inducing DM in Wistar rats using alloxan. The research aims to explore the potential of these plants not only in mitigating the metabolic effects of DM but also in preserving cognitive functions that are often compromised in diabetic conditions.

## Materials and methods

Preparation of extracts

EA and ZJ leaves, harvested locally, were subjected to coarse grinding post-drying. The quality and purity of the resultant crude medicinal product adhered strictly to the standards set by the Indian Pharmacopoeia of 1996. The powdered material was stored in spherical-bottomed glass bottles. Each sample received 3 L of 95% ethanol and underwent a four-hour reflux process, repeated twice. Following filtration, the extracts were concentrated using a rotary evaporator under vacuum at 50°C until dryness was achieved. The final yields of the alcohol-extracted substances from ZJ and EA were quantified, resulting in 31.24% and 29.37% (w/w), respectively.

Ethical considerations

The study on using alloxan monohydrate to make Wistar rats diabetic was done in a way that was completely ethical. The Institutional Animal Ethics Committee (IAEC) of Mamata Medical College gave their approval under protocol number 01/IAEC/MMC. The study adhered to national and international guidelines for humane treatment, ensuring minimal pain and distress for the animals. Rats were housed in controlled conditions and monitored regularly for their well-being. Anaesthesia and analgesia were used during potentially painful procedures to minimize suffering. The health status of the rats was closely monitored post-DM induction, with immediate intervention provided for severe hypoglycemia or other adverse effects. Euthanasia, when necessary, was performed using humane methods. The study was designed in accordance with the principles of reduction, refinement, and replacement (3Rs) to minimize animal use and suffering. Scientific justification for animal use was provided, and detailed records were maintained for all procedures and observations. Finally, the disposal of animal carcasses was conducted in a safe and ethical manner, following institutional and environmental guidelines.

Animal diet

The composition of commercially available food pellets for rats is designed to provide a balanced diet that meets their nutritional needs. Typically, these pellets contain about 20-25% protein, sourced from ingredients like soybean meal, fish meal, or casein. Carbohydrates make up approximately 50-60% of the diet and are primarily derived from grains such as corn, wheat, or oats. Fats account for around 4-5% and are usually obtained from vegetable oils or animal fats. Fiber content is about 3-8%, coming from sources like alfalfa meal, cellulose, or wheat bran. Additionally, a comprehensive mix of vitamins and minerals is included to ensure the rats receive all essential micronutrients. The moisture content of the pellets is typically kept below 10%. The exact composition of these pellets can vary depending on the manufacturer and may be tailored for specific research purposes, such as high-fat diets for obesity studies or diets with added compounds for pharmacological or nutritional research.

Animals and DM induction

Wistar rats (weighing between 200 and 250 grams) were given an intraperitoneal injection of 120 mg/kg of alloxan monohydrate dissolved in 0.9% w/v cold normal saline after not eating or drinking for 12 hours. This made the rats diabetic [7-13]. To prevent hypoglycemia, the rats were then provided with bottles of 10% glucose solution in their cages for the following 24 hours. Fasting blood glucose levels were measured 72 hours after the injection. Animals that did not develop glucose levels exceeding 200 mg/dL were excluded from the study. Forty-two animals (n=42) were selected for the diabetic group, and six animals (n=6) were in the non-diabetic group. Group 1 served as the normal control and was treated with 1 mL of distilled water orally administered using a feeding tube. Groups 2-8 had alloxan-induced DM and received 1 mL of distilled water orally every morning. The animals were given the extracts orally once every morning through compulsory intubations before meals. The treatment continued for 14 consecutive days. The inclusion criteria for the rats were carefully established to ensure the consistency and reliability of the results. The rats selected were within a specific age range and had a similar weight range to minimize variability due to size. Additionally, only Wistar rats were used to maintain consistency in the experimental model. Prior to the study, the rats underwent a thorough veterinary examination to assess their health status, ensuring that they were in good health with no signs of disease or injury. Furthermore, the rats were screened to ensure that they were free from any other comorbidities that could potentially confound the study results. This careful selection and screening process was crucial to the integrity of the study. Throughout the duration of the study (35 days), these rats enjoyed unrestricted access to both commercial pellet food and water. A solution containing 0.1 gram of alloxan monohydrate in saline was carefully prepared in order to induce DM. Subsequently, each rat underwent an intraperitoneal injection of 150 mg/kg of alloxan monohydrate.

Experimental design

After a two-week acclimatisation period, the rats were randomly divided into eight groups (n=6 per group): (1) non-diabetic control receiving 0.5 mL of distilled water; (2) diabetic control induced by alloxan; (3) diabetic treated with glibenclamide; (4-7) diabetic groups receiving varying dosages of EA and ZJ extracts (250 mg/kg and 500 mg/kg); and (8) a combination treatment group of EA and ZJ (low dose). Dosages were determined based on acute oral toxicity studies, with each administered in 0.5 mL of distilled water. Body weights were recorded at the start and on days 7, 14, and 21. Blood glucose levels were monitored using a glucometer prior to and following alloxan treatment, confirming DM in groups 2-8. Each group underwent a 21-day treatment period. To ensure uniformity in the study points, several measures were implemented. All body weight measurements were taken using the same calibrated scale, and blood glucose levels were monitored using the same model of glucometer for all groups, minimizing variability in measurement tools. Measurements were taken at consistent times of day to account for any diurnal variations that might affect the results. Each group underwent an identical 21-day treatment period, ensuring equal exposure to their respective treatments. Randomization was employed to assign animals to each group, reducing the risk of selection bias and ensuring comparability at the start of the experiment. Wherever possible, researchers conducting the measurements were blinded to the group assignments to prevent any bias in data collection. These measures were taken to maintain uniformity in the study points and ensure that any observed differences between groups were attributable to the treatments rather than experimental inconsistencies.

Morris water maze (MWM) test

The MWM test was employed to evaluate the rats' learning and spatial memory capabilities. The test involved a circular pool (1.5 meters in diameter and 60 cm high) with murky water (made opaque with milk), containing a hidden escape platform submerged 1 cm below the surface. The pool was divided into four quadrants, with the platform placed centrally in one. The rats underwent training over three days, with three trials per day and a five-minute interval between each. Each trial allowed 120 seconds for the rat to find the platform, with guidance provided if needed. Rats rested for 30 seconds on the platform post-trial. Each trial commenced from the point farthest from the platform. The time taken by each rat to locate the hidden platform was recorded as transfer/escape latency. On the fourth day, the platform was removed, and the rats were permitted to swim freely. The duration spent in the target quadrant, previously housing the platform, served as a metric for memory assessment [14].

Statistics

The results of each experiment were articulated in terms of mean and standard deviation, providing a concise yet comprehensive statistical representation. For the purpose of statistical analysis, the data was rigorously processed using IBM SPSS Statistics for Windows, V. 27.0 (IBM Corp., Armonk, NY). A one-way analysis of variance (ANOVA) was employed to discern the statistical significance of the findings, with a significance level set at p<0.01. This robust analytical approach ensured a thorough evaluation of the experimental data, allowing for precise and reliable interpretations of the outcomes.

## Results

Antidiabetic activity

**Figure 1:**
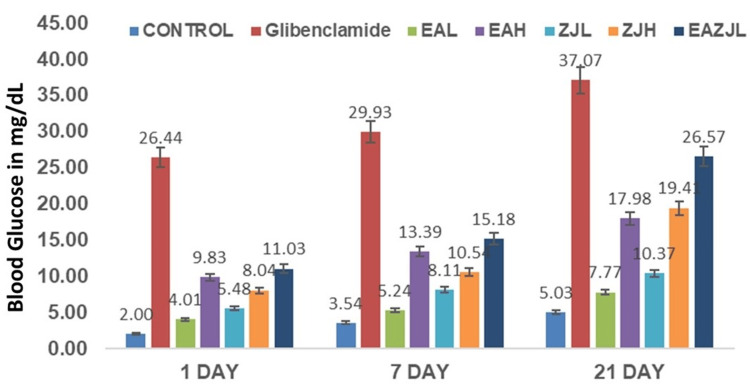
Antidiabetic activity Change in glucose levels over three time points: one day, seven days, and 21 days post-treatment. Each bar represents a different treatment group as follows: CONTROL (untreated), glibenclamide (a standard antidiabetic drug), EAL, EAH, ZJL, ZJH, and EAZJL. Error bars indicate the standard error of the mean. Statistical significance is denoted by p-values, with a p-value of <0.05 considered statistically significant EAL: low-dose *Eclipta alba*; EAH: high-dose *Eclipta alba*; ZJL: low-dose *Ziziphus jujuba*; ZJH: high-dose *Ziziphus jujuba*; EAZJL: low-dose *Eclipta alba* and *Ziziphus jujuba*

**Figure 2:**
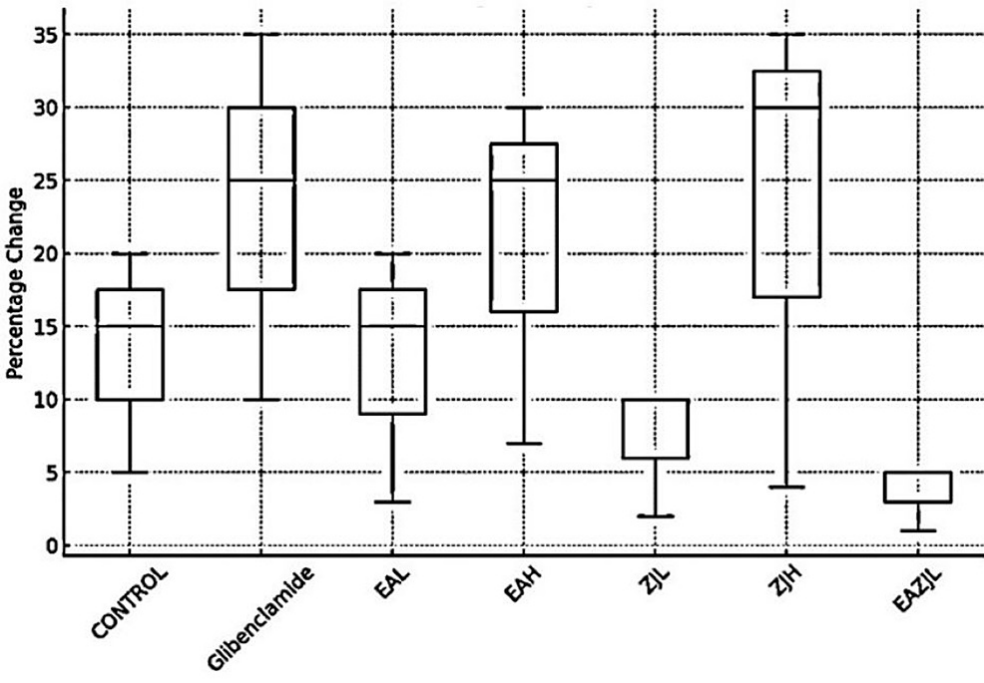
Antidiabetic activity. The box plot represents the percentage change in glucose levels among various treatment groups EAL: low-dose *Eclipta alba*; EAH: high-dose *Eclipta alba*; ZJL: low-dose *Ziziphus jujuba*; ZJH: high-dose *Ziziphus jujuba*; EAZJL: low-dose *Eclipta alba* and *Ziziphus jujuba*

Six days after alloxan administration, rats with DM exhibited significantly elevated blood glucose levels compared to the control group. The initial dose of treatment led to a reduction in the measured intervals. Specifically, the groups treated with high-dose EA (EAH), low-dose EA and ZJ (EAZJL), and glibenclamide showed remarkable peak reductions in blood glucose levels after one day, amounting to 9.83%, 11.03%, and 26.44%, respectively. At the end of the chronic treatment period (21 days), the groups administered EAH, low-dose ZJ (ZJL), high-dose ZJ (ZJH), low-dose EA and ZJ (EAZJL), and glibenclamide demonstrated decreases in blood glucose levels from their initial values by 17.98%, 10.37%, 19.41%, 26.57%, and 37.07%, respectively (Figure 1, Figure 2).

MWM test

**Figure 3:**
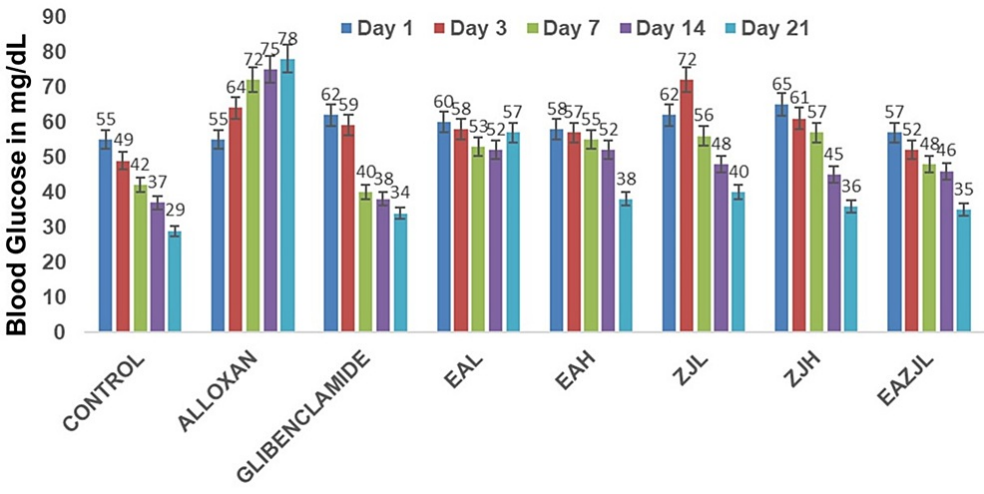
Learning and spatial memory abilities The latency period (in seconds) over a 21-day observation for different treatment groups, relative to the untreated CONTROL group. The bars are annotated for latency periods recorded on day 1, day 3, day 7, day 14, and day 21. n=6, mean±SEM, p<0.01 ALLOXA: the group treated exclusively with alloxan; GLIBENCLAMIDE: used as a benchmark antidiabetic medication; EAL: low-dose *Eclipta alba*; EAH: high-dose *Eclipta alba*; ZJL: low-dose *Ziziphus jujuba*; ZJH: high-dose *Ziziphus jujuba*; EAZJL: low-dose *Eclipta alba* and *Ziziphus jujuba*

**Figure 4:**
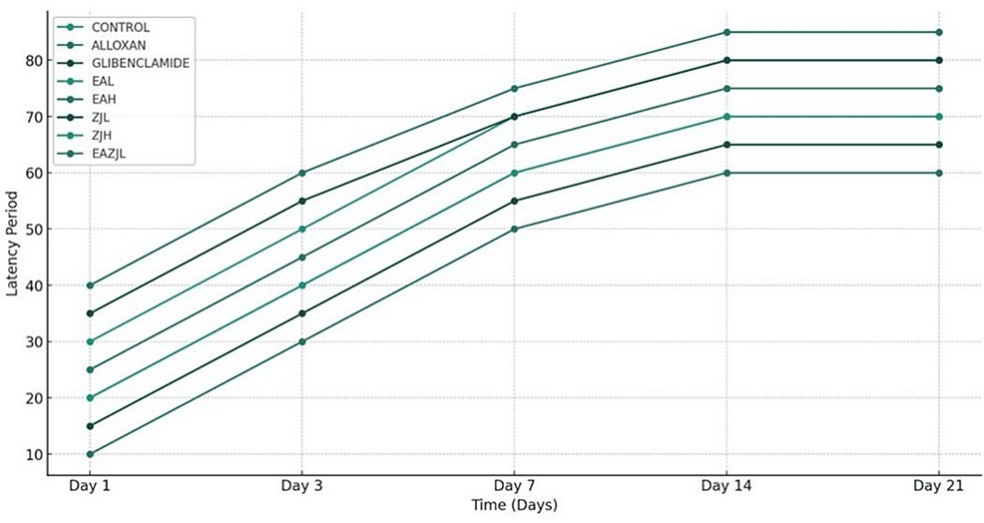
The latency period (in seconds) over 21-day observations EAL: low-dose *Eclipta alba*; EAH: high-dose *Eclipta alba*; ZJL: low-dose *Ziziphus jujuba*; ZJH: high-dose *Ziziphus jujuba*; EAZJL: low-dose *Eclipta alba* and *Ziziphus jujuba*

The MWM was used to assess the learning and spatial memory abilities of rats, and it was done as per the previously described protocol [14]. In the MWM test, diabetic rats did not perform as well as control rats. Furthermore, animals in the DM group found the hidden platform significantly later (transfer latency) than animals in the other treatment groups (p<0.01) (Figure 2). Nonetheless, notable distinctions exist across the EA, ZJ, and additional treatment cohorts, indicating the efficacy of EA and ZJ interventions in augmenting cognitive function under DM-induced circumstances (Figure 3, Figure 4).

## Discussion

Extensive literature suggests that the central nervous system (CNS) is vulnerable to the adverse effects of DM, with the aging brain being particularly susceptible to the harmful impacts of DM-related metabolic disturbances. Research has consistently linked hyperglycemia-induced and lipid dysregulation-induced neuronal damage to cognitive impairments in DM [3,15,16]. In addition, insulin deficiencies and changes can cause innate inflammatory responses that hurt synaptogenesis and cause neurons to die [3].

Over the past decade, advancements in understanding CNS functioning have opened avenues for manipulating CNS activities using advanced psychotropic medications. These developments have raised the potential for enhancing cognitive functions, such as learning and memory, in individuals experiencing cognitive decline.

A recent study investigated how ZJ fruit extract shields PC12 cells from glucose-induced damage when used to study diabetic neuropathy in the lab. The study found that high glucose levels significantly reduced cell viability and increased oxidative stress and apoptosis in these cells. However, treatment with ZJ extract effectively countered these detrimental effects, suggesting its potential in mitigating diabetic neuropathy [17]. In a new study looking into how ZJ might affect Alzheimer's disease (AD), the extract made memory and learning better in rats that had cognitive problems caused by scopolamine. It also reversed brain oxidative stress, inflammation, and apoptosis. These findings support ZJ's traditional use in treating dementia and suggest its potential as an alternative treatment for AD [18]. A previous study investigated the impact of ZJ and its yeast-fermented variant (ZJ-Y) on cognitive deficits in a mouse model of AD. To induce AD-like symptoms, we administered amyloid beta 25-35 (Aβ25-35) to the mice. Our behavioral assessments indicated that both ZJ and ZJ-Y markedly ameliorated the cognitive impairments provoked by Aβ25-35. It is important to note that ZJ-Y was better at lowering oxidative stress markers like malondialdehyde and nitric oxide. This suggests that it has a strong protective effect against the cognitive decline and memory loss that come with AD [19].

In a study on type 2 DM (T2DM) patients, the impact of the sulfonylurea drug glibenclamide on insulin secretion was evaluated across different blood glucose levels. Patients underwent glucose clamps at varying levels after treatment with either glibenclamide or a placebo. Results indicated a significant increase in insulin secretion with glibenclamide, especially at lower glucose concentrations. This suggests that while glibenclamide is effective in stimulating insulin release, it also poses a heightened risk of hypoglycemia, particularly in tight glucose control scenarios [20]. EA, in particular, has been reported to inhibit adipocyte growth and alter AKT signaling, leading to increased peripheral glucose uptake [13].

Yazdanpanah et al. evaluated the impact of ZJ infusion on lipid profiles, glycemic control, and antioxidant status in T2DM patients. In a randomized trial, 116 participants were divided into two groups, with one consuming a balanced diet and the other adding a ZJ fruit infusion to their diet for 12 weeks. The ZJ fruit group showed significant improvements in glycosylated hemoglobin, total cholesterol, triglycerides, and low-density lipoprotein (LDL) cholesterol levels compared to the control group. These findings indicate that ZJ fruit infusion has a beneficial effect on blood sugar regulation and lipid profiles in T2DM [21].

Similar to oral hypoglycemics, many plants exhibit hypoglycemic effects through insulin release and modulation of AKT signaling, with subsequent improvement in peripheral glucose uptake. The leaf extracts of EA and ZJ are posited to aid in insulin sensitization and metabolic function restoration [13].

In the study, enhanced spatial learning and memory were indicated by longer transfer latencies in the MWM test with EA and ZJ extracts. This cognitive improvement is often linked to the nootropic effects of certain plants, which may be attributed to their hypoglycemic activities [22]. In terms of cognitive and behavioral parameters, the EA and ZJ groups significantly outperformed the diabetic control groups, with the latter showing diminished spatial learning and memory [23-28].

Strengths of the study

Our study presents several key strengths that enhance its scientific contribution. Primarily, it targets the critical yet under-explored issue of cognitive impairment associated with DM, using a well-established Wistar rat model to provide a reliable framework for investigation. The meticulous division into eight distinct experimental and control groups, including a standard pharmacological treatment for comparison, allows for a robust evaluation of the therapeutic effects. The inclusion of both short-term (seven days) and long-term (21 days) treatment durations enables an in-depth analysis of the potential lasting impacts of the interventions. Quantitative assessments through blood glucose monitoring, coupled with qualitative evaluations using the MWM test, provide a comprehensive view of the effects on both physiological and cognitive dimensions. The demonstration of statistical significance in the results further reinforces the validity of the conclusions drawn. Additionally, the study suggests the hypoglycemic and cognitive enhancement properties of ZJ and EA extracts, pointing to their potential as nootropic agents and expanding the scope of natural phytochemical research in managing chronic diseases and related neurocognitive disorders.

Limitations of the study

The applicability to human conditions might be constrained by the inherent biological disparities between rats, the chosen animal model, and humans, warranting cautious extrapolation. The study's temporal scope, particularly the 21-day treatment period, raises questions about the durability and long-term implications of the observed cognitive enhancements in the context of the chronic nature of DM. Furthermore, the lack of in-depth exploration into the molecular mechanisms underlying the effects of ZJ and EA extracts, alongside the absence of a diabetic control group treated with a neutral substance, curtails the depth of the study's scientific rigor. While the focus on cognitive improvements is noteworthy, a more comprehensive examination of multi-systemic effects would enhance the study's holistic relevance. Additionally, a thorough presentation of statistical results and ethical considerations would contribute to the transparency and interpretability of the findings. Notably, a more robust comparison with the standard antidiabetic drug glibenclamide could fortify the argument for the therapeutic potential of ZJ and EA extracts.

## Conclusions

Drawing from the data presented, it can be concluded that the administration of EA and ZJ appears to facilitate an improvement in cognitive performance in diabetic subjects. The decrease in latency times throughout the study's timeline, which demonstrates this improvement in cognitive function, emphasizes the potential of EA and ZJ as therapeutic agents. In the future, researchers should try to figure out how these substances improve cognitive function. This will help in the creation of targeted treatments for cognitive problems linked to DM.
